# Cerebral venous sinus thrombosis in patients with inflammatory bowel disease: a retrospective study

**DOI:** 10.1038/s41598-021-96541-y

**Published:** 2021-08-20

**Authors:** Wang Shujun, Zhang Huijie, Bai Xia, Wang Hongjian

**Affiliations:** Department of Gastroenterology, The First Affiliated Hospital of Zhengzhou University, No.1 Jianshe East Road, Zhengzhou, 450052 People’s Republic of China

**Keywords:** Gastroenterology, Risk factors

## Abstract

Cerebral venous sinus thrombosis (CVST) is a rare and devastating complication of inflammatory bowel disease (IBD). Early diagnosis and prompt treatment could improve prognosis. The aim of our study was to investigate the clinical data and predictive factors of inflammatory bowel disease in patients with a diagnosis of CVST. All IBD patient data were collected from July 2013 and September 2020. Clinical data, predictive factors and prognosis were compared between IBD patients with CVST and the IBD control group. The incidence of CVST in our study was 0.48%. The mean age of IBD patients with CVST was 34.9 years. The average duration of IBD was 4 years when cerebrovascular events occurred. The clinical presentation of CVST included headache (73.1%), vomiting (30.8%), limb dysmetria (26.9%), speech impairment (11.5%), blurred vision (7.7%), epileptic seizures (7.7%) and drowsiness (3.8%). The most common location for CVST was the transverse sinus (61.5%) followed by the superior sagittal sinus (30.8%). Anaemia, low albumin and elevated d-dimer were independent predictors of CVST in patients with IBD. Anticoagulation therapy was effective. The prognosis of IBD patients with CVST was worse than that of IBD patients without CVST. Early identification of the risk and clinical features of CVST in IBD patients is important. Prompt antithrombotic therapy is a safe and effective treatment.

## Introduction

Inflammatory bowel disease (IBD) is chronic inflammation of the digestive tube with unknown aetiology. Ulcerative colitis (UC) and Crohn’s disease (CD) are two main types of IBD^1^. In addition to digestive system lesions, IBD is also associated with an abnormal coagulable state. The risk of thrombosis in IBD is increased three- to fourfold compared with that of the general population^2^. Deep venous thrombosis and pulmonary embolism (PE) have been well recognized. Cerebral venous sinus thrombosis (CVST) is a serious complication of IBD with high mortality. The reported incidence of CVST in IBD varies between 0.5 and 6.7%^3^. Clinical symptoms of CVST could vary from headaches to coma, thus making diagnosis difficult. Due to variable clinical manifestations and low incidence, it could be misdiagnosed by physicians.

CVST is suspected to be a consequence of the hypercoagulable state occurring during IBD disease relapse. Several risk factors for vascular thrombosis have been reported for IBD. Smoking, bowel operation history, low haemoglobin, low platelet count and low albumin levels might contribute to thrombosis^4^. Given that the recognition of inflammation can activate coagulation systems, disease activity contributes to coagulopathy^5^. Episodes of intestinal inflammation induce the expression of inflammatory cytokines and the development of a prothrombotic state. IBD treatments, such as steroids^6^, are also associated with CVST. Precise risk factors for CVST remains unclear.

To the best of our knowledge, only a few small sample studies of IBD patients with CVST have been published. Most studies about CVST are case report. CVST often occur during disease flare. Nevertheless, some case reports have shown that CVST occurs during disease remission^7^. A better understanding of the risk factors and clinical manifestations of CVST may assist with early diagnosis and treatment; thus, the prognosis could be improved. This study aimed to investigate predictive factors and clinical features of cerebral vascular thrombosis in patients with IBD.

## Results

### Prevalence of CVST

A total of 5368 patients with IBD were reviewed. Of these IBD patients, 26 patients (n = 20 for UC and n = 6 for CD) were diagnosed with CVST. The incidence of CVST in IBD was 0.48% (26/5368).

### Characteristics of patients with CVST

Patient demographics and underlying diseases are described in Table 1. The mean age of IBD patients with CVST was 34.9 years. The median time interval was 4 years (range: 2 years–6.9 years). The study population included 17 males and 9 females. Most CVST events occurred during IBD disease flares (96.2%), and only 1 patient (3.8%) experienced CVST events during disease remission. Moderate (42.3%) to severe (38.5%) disease was detected in most patients. Laboratory characteristics before CVST and medication history within 6 months were recorded (Tables 2 and 3). No significant differences in sex, age, disease duration, postoperative history, Montreal disease classification, or disease severity were noted between the CVST group and IBD controls.

**Table 1:** Demographic data of IBD patients.

Variables	CVST group 26 patients	Non-CVST group 104 patients	Total 130 patients	*P-*value
Age	34.9 ± 15	38.7 ± 10.3	37.9 ± 11.4	0.127
Male, (%)	17 (65.4)	62 (69.6)	79 (60.8%)	0.59
Disease duration (years)	4 (2–6.9)	5 (2.7–7.3)	4 (2–7)	0.304
VTE history	5 (19.2)	2 (1.9)	7 (5.4)	0.004
Diabetes mellitus	3 (11.5)	3 (2.9)	6 (4.6)	0.094
Atrial fibrillation	4 (15.4)	3 (2.9)	7 (5.4)	0.029
Operation history	4 (15.4)	35 (33.7)	39 (30)	0.094
Smoking	14 (53.8)	18 (17.3)	32 (24.6)	0.001
Alcohol consumption	8 (30.8)	9 (8.7)	17 (13.1)	0.006
**Severity**				0.45
Severe	10 (38.5)	27 (26)	37 (28.5)	
Moderate	11 (42.3)	42 (40.4)	53 (40.8)	
Mild	4 (15.4)	30 (28.8)	34 (26.2)	
Remission	1 (3.8)	5 (4.8)	7 (5.4)	

Values are presented as number (%). VTE, venous thromboembolism.

**Table 2:** Laboratory characteristics of IBD patients with and without CVST.

Variable	CVST group	Non-CVST group	Total	*P-*value
Hemoglobin (g/L)	88 (81.83–93)	104.5 (92–123)	98.60(89–118)	< 0.001
Platelets (× 10^9^/L)	302.5 ± 138.53	286.42 ± 103.56	289.64 ± 111	0.583
Albumin (g/L)	27.35 ± 4.54	34.00 ± 5.53	32.67 ± 6.0	< 0.001
LDL-C (mmol/L)	2.09 (1.47–2.84)	2.08 (1.71–2.65)	2.08(1.68–2.70)	0.852
hsCRP (mg/L)	23.51(7.75–31.80)	21.4 (7.42–53.2)	21.73(7.74–49.91)	0.673
d-dimer (mg/L)	1.19 (0.26–2.9)	0.2 (0.08–0.42)	0.25 (0.09–0.62)	< 0.001

For CVST group, laboratory tests were collected before the event. For control group, laboratory tests were collected at the time of admission.

**Table 3:** Medication history of IBD cohort and outcome of patients.

	CVST group	Non-CVST group	Total	*P-*value
5-ASA	20 (76.9)	70 (67.3)	90	0.342
Steroid use	22 (84.6)	54 (51.9)	76	0.002
Azathioprine	12 (46.2)	46 (44.2)	58	0.86
Infliximab	6 (23.1)	16 (15.4)	22	0.384
Outcome				0.029
Deteriorate	4 (15.4)	3 (2.9)	7 (5.4)	
Improved	22 (84.6)	101 (97.1)	123 (94.6)	

Disease outcome included both IBD and CVST.

Neurological symptoms of CVST included persistent headache (73.1%), vomiting (30.8%), limb dysmetria (26.9%), speech impairment (11.5%), blurred vision (7.7%), epileptic seizures (7.7%) and drowsiness (3.8%). CVST was diagnosed with cranial magnetic resonance imaging (MRI) and magnetic resonance venography (MRV) in 23 patients and with CT in 3 patients. The most common location for CVST was the transverse sinus (61.5%) followed by the superior sagittal sinus (30.8%), sigmoid sinus (23.1%) and straight sinus (7.7%). Infarction was found in 15.4% patients. A total of 104 IBD control patients (80 UC patients and 24 CD patients) were included in the analysis.

### Factors predictive of CVST in patients

Multivariate analysis revealed that anaemia, low albumin and elevated d-dimer were independent predictors of CVST. Smoking, alcohol consumption, history of thromboembolic disease, atrial fibrillation and steroid usage were not significantly associated with CVST in the multivariate analysis despite being significant in the univariate analysis (Table 4).

**Table 4:** Multivariate analysis for predictive factors of CVST in patients with IBD.

	OR	95% C.I	*P-*value
VTE history	3.24	0.407–25.799	0.267
Atrial fibrillation	1.884	0.12–29.621	0.652
Smoking	2.634	0.51–13.589	0.247
Alcohol consumption	4.681	0.726–30.167	0.104
Hemoglobin	0.951	0.907–0.998	0.042
Albumin	0.793	0.668–0.943	0.008
d-dimer	1.301	1.02–1.661	0.034
Steroid use	0.84	0.171–4.138	0.831

### CVST and treatment response

After the diagnosis of CVST, 23 patients were treated with low-molecular-weight heparin (LMWH) and warfarin. Two patients received novel oral anticoagulants. One patient received aspirin and clopidogrel treatment. No patient suffered massive haemorrhage after antithrombotic treatment. Disease outcome included both IBD and CVST. The evolution was favourable in 22 (84.6%) patients and fatal in 4 (15.4%) patients following significant cerebral oedema. Two patients developed infarct progression. The mortality rate of IBD patients with CVST was significantly greater than that of IBD patients without CVST (*p* < 0.05).

## Discussion

To the best of our knowledge, this is the largest clinical retrospective sample study to investigate IBD and CVST to date. CVST has been reported as an uncommon and devastating complication of IBD. The incidence of CVST in our study was 0.48%, which is slightly lower than that noted in a previous study^3^. The difference might be due to different races and nationalities. CVST is a haemostasis disorder. A previous study also showed a reduced risk of VTE in general East Asian populations and hospitalized IBD patients compared with those in Western countries^4^. In addition, given the nature of a retrospective study, patients underwent cerebral image examination only when they exhibited neurological symptoms. Thus, the actual incidence of CVST in IBD might be underestimated.

In our study, CVST often occurred during acute IBD in rare conditions during remission. Case reports also showed occasional events during IBD remission^7^. Males accounted for the majority of patients (65.4%). The mean age of CVST with IBD was 34.9 years, which is lower than the reported average age of 40.7 years in CVST patients without IBD^8^. CVST symptoms were variable and easily ignored. Headache was the most frequent symptom. The transverse sinus and superior sagittal sinus were frequently involved. This finding was consistent with that reported in previous studies^7,9,10^.

Anaemia, low albumin and elevated d-dimer levels were predictive factors for CVST in our study. Previous studies also showed that anaemia and coagulation abnormalities were frequent risk factors for CVST in most reports^9,10^. Elevated d-dimer values were an independent risk factor for thromboembolism in IBD. A case report also demonstrated the predictive value of d-dimer in CVST^11,12^. The role of medical treatment for IBD is controversial. Glucocorticoids were reported to prompt thrombosis by activating coagulation factors and were independent factors for thrombosis in some reports^6,13^. Scholars also had different opinion^14^. The use of steroids was more commonly observed in CVST patients in our study. However, multivariate analysis showed that steroids might not be independent risk factors for CVST. Steroids could be a potential cause of VET but not CVST. Further studies are needed to evaluate the role of steroids in CVST. Given their anti-inflammatory properties, thiopurines and antitumour necrosis factor (TNF) might lower the risk of thrombosis events^15^, whereas other studies documented that high doses of IFX might contribute to CVST^16^. In our study, treatment of IBD did not increase the risk of CVST. The identification of potential predictive factors is important given that specific therapeutic measures and prophylactic anticoagulation to prevent CVST events can reduce mortality and disability.

Treatment of IBD with CVST follows the same regimen as that administered to patients without IBD. The first-line treatment for CVST is adjusted-dose low-molecular-weight heparin (LMWH)^3^. Novel oral anticoagulants are also recommended given their effectiveness and safety in treating cerebral venous thrombosis^17^. Our study showed that antithromboembolism therapy was safe. Dose-weighed LMWH did not increase the risk of massive bleeding. In addition, 2 patients treated with novel oral anticoagulants also achieved good curative effects.

The prognosis of IBD with CVST was worse than that without CVST. A previous study showed that the mortality rate of IBD patients with CVST could reach up to 25%^18^. Timely diagnosis and treatment could result in a better prognosis^10^. For IBD patients with a high risk of CVST, prophylactic anticoagulation with heparin or low-molecular-weight heparin might decrease risk and mortality rates even in IBD patients complicated with CVST^9^. In addition, a study also showed that prophylaxis for thrombosis in IBD patients was safe and should be recommended^19–21^. However, due to the unawareness of the necessity of anticoagulation therapy, the patients in this study were not on heparin prophylaxis.

Our study had several limitations. First, it is a single-centre retrospective study with limited power to detect significant clinical features. The selection of controls could introduce unintentional bias. Second, endoscopic activity plays an important role in assessing disease course. Since we did not have access to complete data on endoscopic activity in the seven-year study period, we used the clinical disease activity instead of endoscopic activity to assess IBD activity. In addition, we did not explore the mechanism of CVST in IBD patients. Multicentre studies are needed to explore preventive and better treatment measures.

In conclusion, CVST was identified in 0.48% of IBD patients in our series. Patients with IBD were younger and more often male compared with those without IBD. Headache was most common symptoms. Anaemia, low levels of albumin and elevated d-dimer were predictive factors. CVST could occur during both the active and remission phases. Acute antithrombosis therapy might improve prognosis.

## Materials and methods

### Study population and data collection

This was a retrospective study conducted in the First Affiliated Hospital of Zhengzhou University between July 2013 and September 2020. The diagnosis of IBD was based on clinical, endoscopic, morphological and histological criteria. Truelove and Witts disease severity classification criteria^22^ and the CD activity index (CDAI)^23^ were used to assess the disease activity of UC and CD, respectively. The diagnosis of CVST was based on clinical symptoms and evidence of thrombosis in cerebral venous sinuses detected by cranial CT, MRI, and magnetic resonance venography^10^. Patients with haematological disease, patients taking contraceptives and IBD patients with incomplete clinical data were excluded. In all CVST patients with IBD, we recorded the type of IBD, including ulcerative colitis (UC) and Crohn’s disease (CD); possible risk factors; clinical manifestations; and radiologic features. Laboratory tests were collected before the event. Medication history within 6 months was also recorded. Controls were randomly selected from patients with a diagnosis of IBD without thrombosis at a ratio of 4:1 (controls:cases). We matched subjects by age, gender and duration. All methods were performed in accordance with relevant guidelines and regulations. Disease activity was assessed before discharge and at each follow-up. This study was approved by the Committee of The First Affiliated Hospital of Zhengzhou University, and all patients signed informed consent forms.

### Statistical analysis

Statistical analysis was performed using SPSS 21.0. Normal distribution of variables was tested. The results are presented as the mean ± standard deviation (SD) or median (25th–75th percentile). Univariate analysis, chi-squared test and Fisher’s exact test were used to compare categorical variables, and unpaired t-test was used for continuous variables. Variables with a *P-*value < 0.05 in the univariate analysis were included in a logistic regression analysis to identify predictive factors associated with the development of CVST.

## Abbreviations

CVST: Cerebral venous sinus thrombosis
IBD: Inflammatory bowel disease
PE: Pulmonary embolism
UC: Ulcerative colitis
CD: Crohn’s disease
SD: Standard deviation
LMWH: Low-molecular-weight heparin
VTE: Venous thromboembolism
LDL-C: Low-density lipoprotein cholesterol
hsCRP: Hypersensitive C-reactive protein
5-ASA: 5-Aminosalicylate
MRI: Magnetic resonance imaging
MRV: Magnetic resonance venography
